# Learning to suppress tremors: a deep reinforcement learning-enabled soft exoskeleton for Parkinson’s patients

**DOI:** 10.3389/frobt.2025.1537470

**Published:** 2025-05-21

**Authors:** Tamás Endrei, Sándor Földi, Ádám Makk, György Cserey

**Affiliations:** ^1^ Faculty of Information Technology and Bionics, Pázmány Péter Catholic University, Budapest, Hungary; ^2^ Jedlik Innovation Ltd., Budapest, Hungary; ^3^ András Pető Faculty, Semmelweis University, Budapest, Hungary

**Keywords:** deep reinforcement learning, soft exoskeleton, Parkinson’s disease, tremor, physics simulation, human–robot interaction

## Abstract

**Introduction:**

Neurological tremors, prevalent among a large population, are one of the most rampant movement disorders. Biomechanical loading and exoskeletons show promise in enhancing patient well-being, but traditional control algorithms limit their efficacy in dynamic movements and personalized interventions. Furthermore, a pressing need exists for more comprehensive and robust validation methods to ensure the effectiveness and generalizability of proposed solutions.

**Methods:**

This paper proposes a physical simulation approach modeling multiple arm joints and tremor propagation. This study also introduces a novel adaptable reinforcement learning environment tailored for disorders with tremors. We present a deep reinforcement learning-based encoder-actor controller for Parkinson’s tremors in various shoulder and elbow joint axes displayed in dynamic movements.

**Results:**

Our findings suggest that such a control strategy offers a viable solution for tremor suppression in real-world scenarios.

**Discussion:**

By overcoming the limitations of traditional control algorithms, this work takes a new step in adapting biomechanical loading into the everyday life of patients. This work also opens avenues for more adaptive and personalized interventions in managing movement disorders.

## 1 Introduction

Neurodegenerative diseases are characterized by the loss of neurons in the central nervous system, which can impact an individual’s quality of life by causing cognitive, motor, or behavioral symptoms Lamptey et al. (2022). The occurrence of these disorders is expected to increase, partly due to the recent growth in the aging population Heemels (2016). Neurological tremors are the most common of the movement disorders Louis et al. (1995), present in multiple neurodegenerative disorders such as essential tremor Deuschl et al. (1998) and Parkinson’s disease Lang and Lozano (1998b), Lang and Lozano (1998a). Tremors can be described as involuntary, oscillating, or rhythmic movements Bhatia et al. (2018). Although these are not life-threatening, movement disorders pose serious difficulties in daily activities, functional disabilities, and social inconvenience, as well as difficulties performing tasks that require fine motor skills for two-thirds of the affected patients Rocon et al. (2007b).

Although there is no cure for neurodegenerative diseases, current treatments aim to alleviate symptoms and enhance patient well-being. Invasive options, such as deep brain stimulation Oliveira et al. (2023); Faraji et al. (2023), neurosurgery Albano et al. (2023), and stem cell therapies Heris et al. (2022), can be effective but often come with high costs and severe side effects. Non-invasive treatments have been explored, ranging from medication Asil et al. (2020) and traditional Chinese therapies Cai et al. (2023) to advanced wearable technologies. These include robotic exoskeletons Rocon et al. (2007a); Herrnstadt and Menon (2016) and soft exoskeletons Skaramagkas et al. (2020); Awantha et al. (2020); Zahedi et al. (2021), as well as functional electrical stimulation (FES) devices Dosen et al. (2014); Jitkritsadakul et al. (2017), which use electrical stimulation. Additionally, afferent neuroprostheses have been developed to stimulate the patient’s central nervous system Pascual-Valdunciel et al. (2020); Dideriksen et al. (2017). Of all the non-invasive treatment options, the use of exoskeletons has been proven to be the most efficient method for the suppression of tremors Lora-Millan et al. (2021).

Wearable exoskeleton research mainly focused on reducing the weight of exoskeletons Yi et al. (2019); Wang et al. (2023) due to their bulk and weight limiting their adoption. Therefore, control algorithms were not the main interest of these studies, which often utilized repetitive control Rocon et al. (2007a), traditional control methods Herrnstadt and Menon (2016); Zhou et al. (2017); Yi et al. (2019); Zahedi et al. (2021), tremor frequency noise filtering Taheri et al. (2013), or equivalent-input-disturbance (EID) tremor suppression Xie et al. (2024). Traditional control methods, though widely used, have significant limitations. They are typically validated on low-degree-of-freedom systems and under static conditions, overlooking tremor propagation and the natural frequencies of voluntary movements. Evaluations often rely on healthy subjects mimicking tremors, which fails to capture the multi-harmonic characteristics of Parkinson’s tremors. Additionally, these methods do not quantify or account for interference with voluntary motion. For dynamic movements, traditional methods require either time-consuming patient-specific training with human-in-the-loop optimization Siviy et al. (2023); Ding et al. (2018) or manual rule design for each activity, limiting the scalability and adoption of wearable robotics Slade et al. (2022). In contrast, recent advances in deep reinforcement learning (DRL) have shown promise in managing stochastic action spaces in robotics Jin et al. (2022); Kaufmann et al. (2023); Haarnoja et al. (2023) and are gaining traction for rehabilitation exoskeletons, as DRL enables simulation-based training without additional patient involvement Luo et al. (2021), Luo et al. (2023), Luo et al. (2024).

**FIGURE 1:**
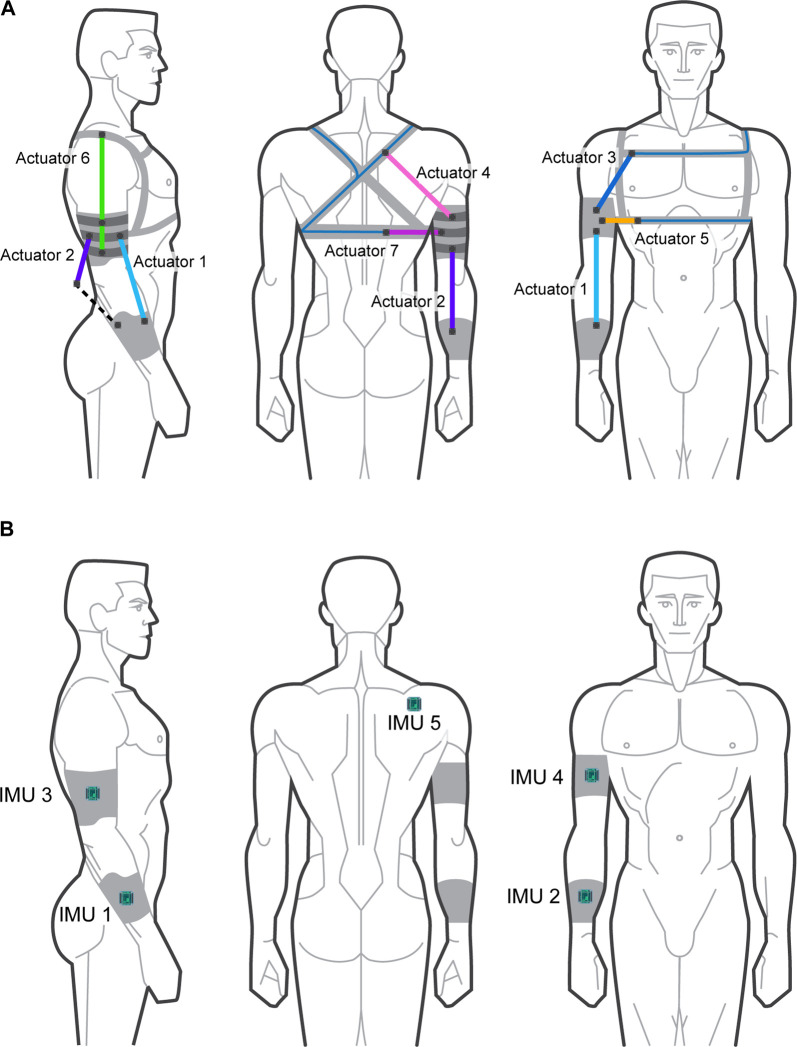
The anatomy of the tremor-suppression exoskeleton and the corresponding inertial measurement unit (IMU) reference movement acquisition system. **(A)** The soft-robotic exoskeleton used in the tremor-suppression simulations. **(B)** The IMU reference movement acquisition system. **(A)** Actuator positions. **(B)** IMU sensor positions.

Therefore, our work aims to incorporate recent advances in DRL and makes the following central contributions to the field of biomechanical loading exoskeletons:

• We create a human–exoskeleton simulation environment that is capable of simulating multiple different dynamic movements, different types of tremors, and human–exoskeleton interactions.
• We propose a model-free deep RL-based tremor-suppression controller capable of suppressing generated tremors across various axes of the shoulder and elbow joints during a multitude of dynamic movements.
• We demonstrate that the soft exoskeleton Figure 1A, coupled with our DRL-based controller, can accurately mitigate the effect of generated tremors.

The result is an intelligent tremor-suppression controller that minimizes its effects on the patient’s original movements and posture, with no additional training required from the patient to adapt to the exoskeleton.

In the following sections, we detail the underlying physical simulation and the DRL framework, describe the experimental setup and evaluation metrics, present our results, and discuss the implications of our approach for future wearable robotics in the treatment of neurodegenerative movement disorders.

## 2 Methods

### 2.1 Tremor-suppression physical simulation

To facilitate the training of a reinforcement learning-based controller, we established a physical simulation environment to ensure a secure and cost-effective learning process. The simulation uses a human torso model with the addition of the right arm, in which the tremors induced will be suppressed. The simulation environment uses the Pybullet physics engine Coumans and Bai (2016) and the Open AI gym Brockman et al. (2016) to create a reinforcement learning environment.

**FIGURE 2:**
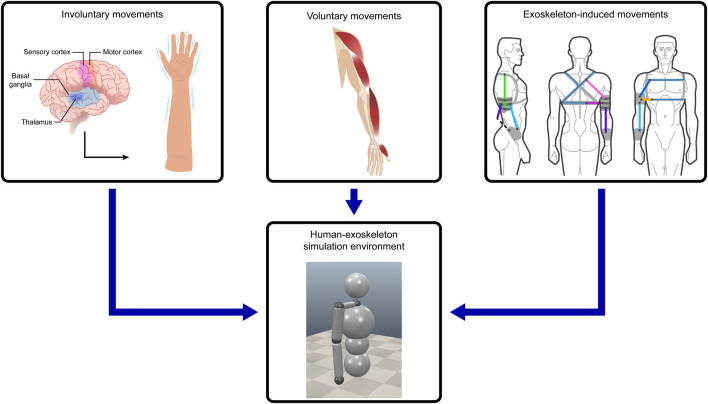
The parts of the simulation. Voluntary movements represent the trajectory of the movement in which involuntary movement tremors are generated, which the exoskeleton tries to suppress using its actuators.

The human–exoskeleton simulation is made up of three distinct parts. The movements were recorded using two Velcro sleeves fixed around the upper and lower arm, with an additional inertial measurement unit (IMU) sensor fixed on the scapula of the right arm, as presented in Figure 1B. These parts of the simulation are illustrated in Figure 2, and described in the following sections.

#### 2.1.1 Acquiring reference movements

The reference movements represent the patients’ voluntary movements, which act as the base trajectory for the environments. In the reference motions, we have recorded four distinct movements: shoulder flexion/extension, shoulder abduction/adduction, elbow flexion/extension, and the external rotation of the shoulder. Two distinct recordings are used for each distinct movement pattern for training to add variability and improve the robustness of the controller.

Of the five IMU sensors this system possessed, IMU 2 and IMU 4 were chosen. From the accelerations and angular accelerations measured, we could approximate the quaternions of the shoulder and elbow joints using an extended Kalman filter Welch and Bishop (1995). Finally, the quaternions were transformed into Euler angles, which were used in the simulation.

Verbal informed consent was exchanged between the authors and the subject when planning, preparing, and executing the IMU measurements.

#### 2.1.2 Generating tremors

Our generated involuntary movements can be described by three attributes: their amplitude, frequency, and the time duration during which the tremor effects are present. The third attribute can be disregarded to ensure a more computationally effective simulation and training of the control. Therefore, tremorous movement parts are present at every simulation time step.

In our simulations, we utilized Parkinson’s disease tremors due to their well-documented and well-understood characteristics. Tremors present in Parkinson’s disease can be described as a second-order non-linear stochastic process Taheri et al. (2013), which can be approximated by the superposition of sine waves Riviere et al. (1997).

In this paper, we approximated these tremors by two sine waves with given parameters based on Taheri et al. (2013). This way, the approximation contains 96.4±1.39% of the original energy of the tremor.

From the two main frequency ranges, we randomly sampled values for both harmonics independent from each other and added Gaussian noise to their sum, which was then consequentially normalized. To ensure a wide range of possible tremor cases, a vector containing the given arm joint axis was used to specify which joint axis was affected by tremors.

Finally, from this created vector, which contains the tremor acceleration values for each joint axis in the arm, we transformed these values into torque values based on the measurements done by Ketteringham et al. (2014).

In tremor instances, where the effect of tremor impacts multiple joint axes, the frequencies are kept the same for all involved axes Davidson and Charles (2017).

### 2.1.3 Defining human–exoskeleton interactions

Our simulation incorporates reference, tremorous, and exoskeleton-induced movements using a position-controlled upper torso model with one tremor-affected arm.

The exoskeleton uses an active control strategy that applies force directly to the arm. The following equations describe the process in which the force is converted into torque values that are used during the training process.

First, we can denote an actuator’s state by knowing the positions of their two ends. We denote these by naming the starting point of the actuator with the number 1 and the endpoint with 2, where the actuator will exert its force and pull towards the start point.

The actuator force is a 3D force whose components are proportional to the angles of displacement that the two points create. With the denoted displacement angles, force components that the actuator creates on the arm at that given position can be calculated using Equations 1–3.

$$F_{x}=\cos\left(atan2\left(P_{2y}-P_{1y},P_{2x}-P_{1x}\right)\right)\cdot F$$ (1)

$$F_{y}=\cos\left(atan2\left(P_{2x}-P_{1x},P_{2y}-P_{1y}\right)\right)\cdot F$$ (2)

$$F_{z}=\cos\left(atan2\left(P_{2z}-P_{1z},P_{2x}-P_{1x}\right)\right)\cdot F$$ (3)

To calculate the torque vectors these forces create, we first calculate the position vectors. These can be calculated with a simple vector subtraction of the point denoting the position of the specific joint (shoulder or elbow) and the endpoint of the actuator.

Finally, the torque values are calculated by the vector product of the force components and the position vectors and summed up for each specific joint axis. The values are then used inside the reinforcement learning environment.

### 2.1.4 The simulation system

**FIGURE 3:**
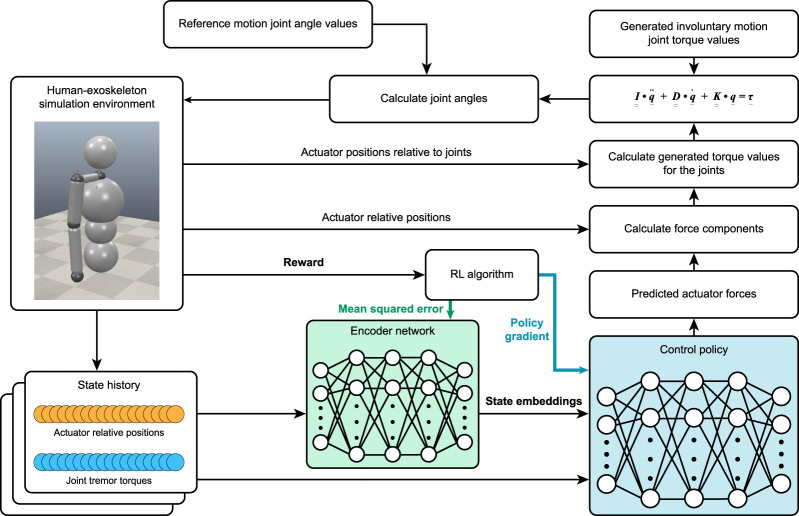
The complete learning process of the control policy. As a deep reinforcement learning agent, we construct our controller as a multilayer perceptron (MLP) neural network. The control policy and encoder networks are updated as described in the TD7 algorithm.

For the control’s learning loop (Figure 3A), reference movements and the joint axes are chosen in which tremors are present. At the beginning of the episode, all actuator forces are set to 0. After summing up the actuator and tremor-generated torque values, Equation 4, a second-order, seven-variable differential equation, is solved (Davidson and Charles, 2017; Corie and Charles, 2019):

$$\underline{\underline{I}}\cdot \ddot{q}+\underline{\underline{D}}\cdot \dot{q}+\underline{\underline{K}}\cdot q=\tau ,$$ (4)

where $q={\left[q_{1},q_{2},q_{3},q_{4},q_{5},q_{6},q_{7}\right]}^{T}$ is the angle of displacement in each joint degree of freedom (DoF). The elements of q represent the following angles: $q_{1}$ : shoulder flexion/extension (SFE), $q_{2}$ : shoulder abduction/adduction (SAA), $q_{3}$ : shoulder external/internal rotation (SEIR), $q_{4}$ : elbow flexion/extension (EFE), $q_{5}$ : forearm pronation/supination (FPS), $q_{6}$ : wrist flexion/extension (WFE), and $q_{7}$ : wrist radial-ulnar deviation (WRUD).

The 7 × 7 matrices present the coupled inertia $\underline{\underline{I}}$ , damping $\underline{\underline{D}}$ and stiffness $\underline{\underline{K}}$ of the mentioned DoF, respectively Davidson and Charles (2017).

Therefore, this takes tremor propagation into account with the inclusion of anatomically coupled properties of the joints. Furthermore, upon closer inspection, we can break down the torque values τ to the following components (Corie and Charles, 2019): $\tau_{I}$ : torque required to perform an intentional task, $\tau_{T}$ : torque generating the tremor, $\tau_{L}$ : task load torque, and $\tau_{G}$ : gravitational torque, $\tau_{O}$ : torque generated by the orthosis (exoskeleton) on the particular joint.

The mentioned components $\tau_{I}$ , $\tau_{L}$ , and $\tau_{G}$ are covered by the reference movement, thus leaving the torque generated by the tremor and the exoskeleton to find the unknown joint angle displacement values in our calculation.

With the calculated joint angle values based on the reference motion recording and tremor–exoskeleton interaction, we simulate one step in our simulation and receive a new state observation.

This new state observation is then propagated through an encoder neural network to further extract hidden information or unrealized correlations in the data, which are then given as the input to the control policy alongside the original observation vector that the encoder received.

For the anatomical properties of the simulation, the joint angle ranges are based on Zwerus et al. (2019) and Gill et al. (2020). The maximum joint torques for the voluntary motion are designed according to Otis et al. (1990) and Günzkofer et al. (2012). The upper and lower arm weight ratios are defined by Plagenhoef et al. (1983).

#### 2.1.5 Dynamics randomization

Although simulation-based training provides a safe and efficient way to train our controller, there is a well-known discrepancy called the sim-to-real gap between the physical and real-world environments.

In order to overcome this obstacle and improve the robustness of our control, we employ dynamics randomization (Sadeghi and Levine, 2016; Tobin et al., 2017; Peng et al., 2018b).

This method randomly samples environmental characteristics from a given uniform distribution (Table 1) at the beginning of each episode. This forces our agent to be more robust against perturbations present in the environmental characteristics and to better adapt to the real-world environment, whose characteristics are expected to be present in the given distribution ranges.

### 2.2 Control algorithm training

In this section, we propose a deep reinforcement learning-based training and testing framework that enables the learning of optimal tremor-suppression strategy.

#### 2.2.1 Reinforcement learning background

Reinforcement learning (RL) is a branch of machine learning that deals with sequential decision-making problems (Sutton and Barto, 2018). The objective is to learn an optimal policy π that enables an agent to maximize its return through interactions with a specified environment. The return, which is described as the discounted cumulative rewards the agent collects, is defined by Equation 5.

$$R_{t}=\overset{T}{\underset{i=t}{\sum }}\gamma^{i-t}\cdot r\left(s_{i},a_{i}\right)$$ (5)

The agent at each discrete time step t , with a corresponding state s∈S , selects an action a∈A with respect to its policy π:S→A , receiving reward r and a new state of the environment $s^{\prime }$ .

Deep reinforcement learning is the combination of deep neural networks with RL, where the policy is represented by a neural network $\pi_{\theta }$ , where θ denotes the weights of the network.

#### 2.2.2 TD7

A popular family of RL methods is actor-critic algorithms, where a policy known as the actor is updated by the deterministic policy gradient algorithm (Silver et al., 2014) Equation 6:

$$\nabla_{\theta }J\left(\theta \right)=E_{s∼p_{\pi }}\left[\nabla_{a}Q^{\pi }\left(s,a\right){|}_{a=\pi \left(s\right)}\nabla_{\theta }\pi_{\theta }\left(s\right)\right]$$ (6)

In Equation 7, $Q^{\pi }\left(s,a\right)$ is known as the critic or value function, which is used to calculate the expected return when performing action a in a given state s following the actor policy π .

$$Q^{\pi }\left(s,a\right)=E_{s_{i}∼p_{\pi },a_{i}∼\pi }\left[R_{t}|s,a\right],$$ (7)

which is commonly updated by temporal difference learning utilizing a secondary target network as described by Mnih et al. (2013) see Equation 8.

$$y=r+\gamma Q_{\theta^{\prime }}\left(s^{\prime },a^{\prime }\right),\;a^{\prime }∼\pi_{\theta^{\prime }}\left(s^{\prime }\right),$$ (8)

where $Q_{\theta^{\prime }}\left(s,a\right)$ is the target critic network, and $\pi_{\theta^{\prime }}$ is the target actor network.

These methods are prone to overestimation errors, whereby, through the function approximation error of the critic, some state-value pairs are overestimated, leading to a sub-optimal policy. The twin delayed deep deterministic policy algorithm (TD3) (Fujimoto et al., 2018) solves function approximation errors by the use of a second critic network and clipped doubled Q-learning (Van Hasselt et al., 2016), as shown in Equation 9.

$$y=r+\gamma \underset{i=1,2}{\min}Q_{\theta_{i}^{\prime }}\left(s^{\prime },\pi_{\theta_{1}}\left(s^{\prime }\right)\right)$$ (9)

In our proposed reinforcement learning-based controller, a state-of-the-art reinforcement learning algorithm called TD7 (Fujimoto et al., 2023), which incorporates the following additions to the TD3 algorithm, is used.

A loss-adjusted prioritized (LAP) (Fujimoto et al., 2020) replay buffer improves the sample efficiency of the algorithm and speeds up training by sampling transition tuples $i≔\left(s,a,r,s^{\prime }\right)$ from which the agent can learn more. The probability of sampling transition i from the replay buffer B sampling is

$$p\left(i\right)=\frac{\max\left(|\delta \left(i\right){|}^{\alpha },1\right)}{\sum_{j\in B}\max\left(|\delta \left(i\right){|}^{\alpha },1\right)},\;where\;\delta \left(i\right)=Q\left(s,a\right)-\left(r+\gamma Q\left(s^{\prime },a^{\prime }\right)\right)$$ (10)

In Equation 10, the level of prioritization is governed by the hyperparameter α .

Behavioral cloning term allows the use of the algorithm in an offline-RL setting Fujimoto and Gu (2021). However, because our task relies on online training, we do not go into depth for this addition.

Policy checkpoints add additional stability toward the training of the agent by selectively employing the best-performing networks, therefore providing stability.

State-action learned embeddings aim to improve the inputs to the actor and critic networks by capturing the relevant underlying structure of the observation space and the transition dynamics present in the environment. Therefore, our network equations can be described by Equation 11 as follows:

$$Q\left(s,a\right)\to Q\left(z^{sa},z^{s},s,a\right),\;\pi \left(s\right)\to \pi \left(z^{s},s\right),$$ (11)

where $z^{s}$ is the state embedding, and $z^{sa}$ refers to the state-action embedding.

The choice of TD7 (Fujimoto et al., 2023) over other widely used reinforcement learning algorithms such as PPO (Schulman et al., 2017), TD3 (Fujimoto et al., 2018), or SAC (Haarnoja et al., 2018) is motivated by several key factors. First, TD7 exhibits significantly improved sample efficiency, often achieving comparable performance to prior methods with only one-tenth of the training time steps. Second, it demonstrates substantially higher performance across standard gym benchmark tasks (Brockman et al., 2016). Finally, TD7 incorporates embeddings that enable the use of larger neural network architectures. A detailed list of hyperparameters with their justifications is provided in the supplementary material.

#### 2.2.3 Observations, actions, and rewards

At each time step t , an observation vector of $o_{t}$ $\in \;R^{80}$ . The observation/state vector is defined by $o=\left\{F_{t-2:t},\tau_{t-2:t},p_{t-1:t}^{a},p_{t-1:t}^{j}\right\}$ , in which F contains the force values of the actuator, τ refers to the tremor torque, $p^{a}$ contain the end position coordinates of the actuators, and $p^{j}$ denotes the coordinate positions of the joints. In this observation vector, all the values are normalized.

For each observation vector, the actor network outputs an action $a_{t}$ $\in \;R^{7}$ in the form of the output force of each actuator. These actions are then converted into the ranges of the actuator forces *F*.

To achieve the complex tremor-suppression behavior of our agent, a densely constructed reward function is utilized. The aim of the control is to suppress tremors to the maximum extent while interfering the least with the voluntary movement of the patient and also utilizing the minimum force required.

Therefore, the reward function consists of five parts: a part accounting for mitigating the tremor torque, a sub-reward accounting for the distortion of the original movement trajectory, a term encouraging tremor reduction across all the affected axes, an actuator smoothness reward, and a reward encouraging the use of minimal actuator force in order to control this tremor torque. This reward is based on the reinforcement learning heuristics of reward shaping (Peng et al., 2018a). The full reward function is written as Equation 12:

$$r_{t}=w_{a}\cdot r_{t}^{a}+w_{\tau }\cdot r_{t}^{\tau }+w_{F}\cdot r_{t}^{F}+w_{as}\cdot r_{t}^{as}+w_{u}\cdot r_{t}^{u}$$ (12)

where $w_{a}$ , $w_{\tau }$ , $w_{F}$ , and $w_{as}$ are the respective weights of the sub-rewards. The values of the weights are $w_{a}=0.5$ , $w_{\tau }=0.9$ , $w_{F}=0.05$ , $w_{as}=0.05$ , and $w_{u}=0.5$ . $F_{s}$ and $F_{e}$ denote the maximum actuator forces possible at the shoulder and elbow actuators.

The tremor axis reward $r_{a}$ aims to encourage control strategies that suppress tremors across all the involved joint axes, as defined in Equation 13:

$$r_{t}^{a}=w_{a}\cdot n_{a}$$ (13)

where $n_{a}$ is the number of axes where generated tremor torques are present.

The torque reward $r_{t}^{\tau }$ enforces the agent to mitigate tremors in all the affected joint axes:

$$r_{t}^{\tau }=\exp\left[\frac{-\sum_{i=1}^{n}\left(\left(\left|\tau_{e}^{i}|-|\tau_{t}^{i}\right|\right)/|\tau_{t}^{i}|+1\right)}{n}\right]$$ (14)

Equation 14 contains the unmitigated original tremor-generated torque values $\tau_{t}$ and the torque values after the exoskeleton has applied its forces $\tau_{e}$ . The equation calculates the tremor suppression on a given joint axis, which is then averaged to be capable of handling tremors affecting multiple joint axes.

The actuator force reward encourages the agent to apply minimal forces with the exoskeleton actuators, reducing energy expenditure, improving efficiency, and preventing damage to the exoskeleton and the patient.

$$r_{t}^{F}=\exp\left[\frac{-\sum_{i}F_{i}}{F_{e}+F_{s}}\right]$$ (15)

In Equation 15, $w_{a}$ is an actuator weight aimed to magnify the learning signal, whose value is dependent on the highest maximum force output and the number of actuators present in the exoskeleton.

The action smoothness reward Equation 16 promotes the use of smooth actuator forces by penalizing the second-order derivatives of the actuator forces:

$$r_{t}^{as}=\frac{1}{N\cdot \frac{F_{e}+F_{s}}{2}}\overset{N}{\underset{i=1}{\sum }}{\left(F_{i}-2\cdot F_{i-1}+F_{i-2}\right)}^{2}$$ (16)

Because exoskeletons can disrupt natural movements, the “unwanted movement” reward Equation 17 is added to ensure smoother, more natural motion, minimizing discomfort and improving efficiency. The reward discourages the control from interfering with the voluntary movement trajectory by penalizing the amount of torque created on non-tremor-affected axes.

$$r_{t}^{u}=\exp\left[\frac{-\sum_{i}\tau_{i}^{u}}{\frac{F_{e}+F_{s}}{2}}\right]$$ (17)

#### 2.2.4 Modifications to handle tremor suppression

Given the diverse nature and precision demands inherent in the tremor-suppression task, the training algorithm has undergone specific modifications to accommodate these challenges.

First, a modification is made to the replay buffer to handle the variance in movement trajectories present in the reference movement. This way, the replay buffer is divided into as many sub-parts as there are reference movements, and then from these sub-buffers, we sample a batch size number of transitions according to prioritized experience replay (Fujimoto et al., 2020). This modification improves robustness because oversampling is avoided even though the different length reference movements create an uneven data distribution in the buffer overall. The sub-buffer also has an increased size to leverage a wider range of possible transitions present to improve the performance of training (Fedus et al., 2020).

The other main modification is regarding the decrease of action and policy noise in the algorithm. This helps by reducing the random space around the agent’s chosen action/policy values, therefore allowing it to learn more fine-tuned control policies. This is crucial because a small change in actuator force can lead to vast differences in the torque created on the human skeleton due to anatomical reasons.

Tremor suppression via exoskeleton requires sophisticated actuation of different motors, where we found that typical white noise exploration added to the chosen actions is not sufficient. Therefore, we replace this common method by adding pink noise (Eberhard et al., 2023) to the actions, improving the agent’s exploration ability and improving action smoothness by incorporating a more correlated noise to the actions.

#### 2.2.5 Training details

The training of the agent is performed in one set of parallel environments, where each represents a reference movement trajectory and a distinctly generated tremor, with the axes defined where tremors are present. The axes in which the tremors are present are constant throughout all the dynamic movements. The agent does not use random state initialization or early termination, but it ensures that the simulated trajectory remains close to the original trajectory by initializing each simulation step from the original value of the reference movement and not the previous simulation step positions. This ensures robustness and boosts performance.

The specifics of the networks and hyperparameter details of the training are found in the supplementary materials.

## 3 Results

To analyze our control algorithm’s performance, a number of numerical tests were conducted to answer the following questions: 1) Can the trained agent suppress the generated tremors across various joints and reference movement? 2) How accurately can it mitigate the effects of tremors, and at what percentage? 3) To what extent are the generated tremor torque values suppressed? 4) How is the original movement trajectory affected by the exoskeleton?

### 3.1 Evaluation of the control policy

**FIGURE 4:**
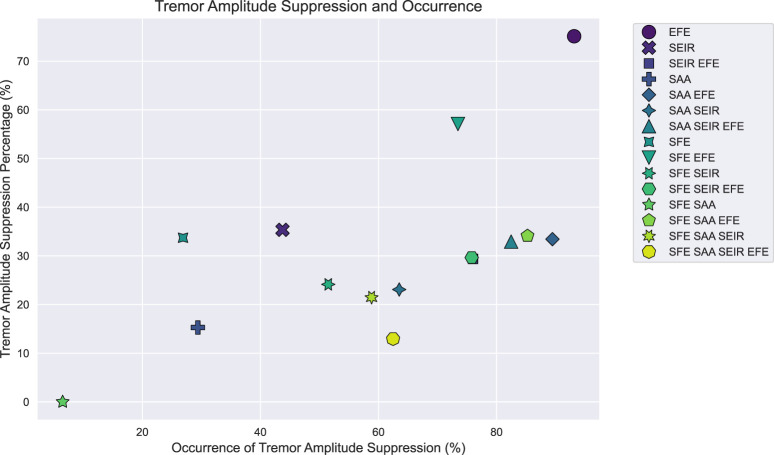
Tremor amplitude suppression values throughout the tremor pairs. Tremor suppression was evaluated for each case over 100 episodes across all reference movements, averaging tremor amplitude suppression and occurrence values. Tremor occurrence indicates the percentage of time steps where the tremor was reduced without disrupting the person’s original trajectory.

The control policy was evaluated through 100 episodes, each of which consisted of an environment with each of the reference movements. The environment characteristics were sampled from a larger dynamics randomization testing range (Table 1) to display the learned controller’s ability to generalize to out-of-distribution cases. The frequency components for the tremors were randomly generated in the specified range in each episode and environment. The control has been trained and evaluated for each possible tremor combination involving the shoulder axes and the elbow extension/flexion axis. The tremor amplitude suppression percentages and the occurrences of tremor suppression without interfering with the original movement trajectory can be seen in Figure 4. The controller effectively suppresses tremors in all but one of the generated tremor pairs, demonstrating a high level of generalizability of the method. In-depth performance data for each of the combinations of the tremor joint axes are presented in the supplementary material.

**TABLE 1:** Dynamic randomization parameter ranges used throughout training and validation. Anatomical matrices represent values in the inertia, damping, and stiffness matrices. Actuator precision accounts for the discrepancy between the commanded and actual force generated by the actuator. Actuator end-point shift refers to actuator sliding due to soft-robotic Velcro changes. Tremor frequencies and amplitude reflect different Parkinson’s patients’ tremor characteristics.

Dynamics parameters	Training range	Testing range
Anatomical matrices	[0.9, 1.1] ⋅ original value	[0.875, 1.125] ⋅ original value
Actuator precision	[0.97, 1.03] ⋅ original value	[0.96, 1.04] ⋅ original value
Actuator end-point shift (in one axis)	[0, 2] cm	[0, 2.5] cm
Tremor first harmonic frequency	[4, 6] Hz	[3.75, 6.25] Hz
Tremor second harmonic frequency	[8, 12] Hz	[7.5, 12.5] Hz
Tremor amplitude	[0.1, 1] ⋅ max value	[0.05, 1.05] ⋅ max value

**FIGURE 5:**
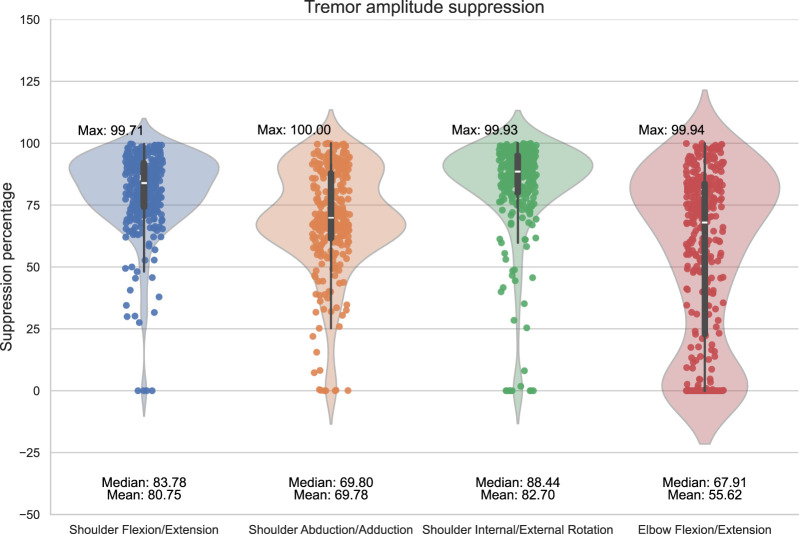
Tremor amplitude suppression values throughout the movements. We display the tremor amplitude suppression values achieved by the exoskeleton throughout the recorded dynamic movement over the time steps of a single episode. The reported statistics are computed over 100 episodes with out-of-training distribution domain randomization.

To further investigate the reference motion-wise performance of the controller, we evaluate a tremor case involving the elbow flexion/extension axis of the arm. The performance of the control can be seen in Figure 5.

These results demonstrate that a single RL-based trained controller can adapt to mitigate tremors regardless of the reference movement. The control also displays high performance, with the maximum tremor amplitude suppression values exceeding 99%. The control also displays a consistent ability to suppress tremors, evident from the median and mean values of Figure 5.

**FIGURE 6:**
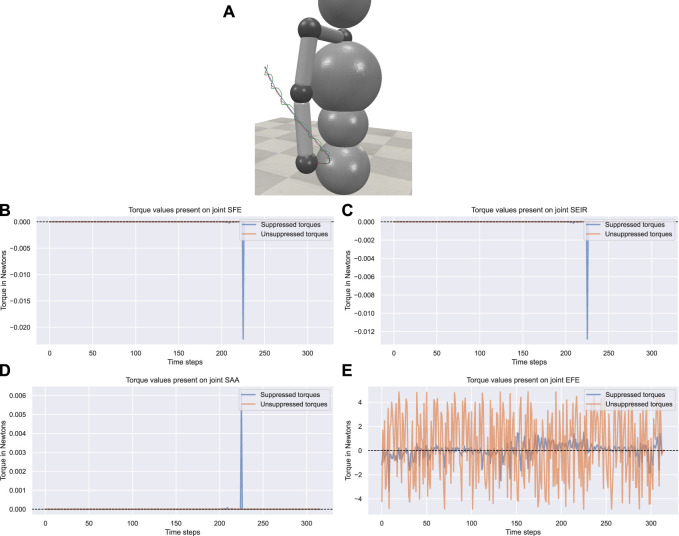
The trajectories of the simulation. **(A)** The trajectories in the 3D simulation environment. Trajectories were recorded during an external rotation movement of the shoulder. Tremors were observed in the flexion/extension axis of the elbow joint. The blue represents the original trajectory, the green represents the trajectory affected by the tremor, and the red represents the exoskeleton-suppressed trajectory. **(B -E)** The suppressed and unsuppressed torque values present at each joint.

The qualitative performance of the controller can be seen in Figure 6A. The figure shows how the original movement trajectory is affected by the exoskeleton. This figure presents additional evidence, as the suppressed movement trajectory consistently maintains a shorter distance from the reference movement’s trajectory when compared to the trajectory affected by tremors.

The torque plots in Figures 6B–D display the controller-created torque present on the joints unaffected by the simulated tremor. The controller’s ideal behavior, which is derived from the torque values achieving the optimal zero generated torque at given time steps, can be seen in the figures. Furthermore, when the torque values are not 0, we can see a tendency in the time steps to minimize this torque and correct the control behavior.

The torque plot in Figure 6E shows the torque created by the controllers present in the joint affected by the simulated tremor. The trained controller effectively suppresses tremors in the involved axis in which tremors are present, although it is prone to producing different torque suppression percentages. The concrete torque suppression values for each tremor pair averaged across the movements are provided in the supplementary material.

## 4 Discussion and limitations

The control of soft-robotic exoskeletons requires real-time decision making on a wide range of stochastic predictors and changing sensory readings in dynamic everyday movements. Furthermore, validation of these control algorithms requires extensive testing to ensure the safety and performance of the control.

This work contains an easily adaptable testing environment for all sorts of neurodegenerative diseases displaying symptoms of tremors, allowing for rapid and cheap testing for new learning-based control methods.

The controller also displays good performance in mitigating tremor torques and amplitudes. However, this performance fluctuates over time steps. The controller’s performance varies between movements and tremor cases. This is due to the exoskeleton structure and also the simulation tremor torque values used. In future studies, exploration of tremor torque ranges is warranted. Furthermore, addressing performance optimization requires a more nuanced understanding of the involved joint axes and their associated characteristics in a given dynamic movement.

From the tremor torque plots, it is evident that further reduction in the tremor amplitude is dependent on how effectively the exoskeleton only exerts forces onto the axes where tremors are present. This could involve revisiting the actuator positions or perhaps using a mixed method of FES and robotic exoskeletons. This approach could minimize tremors in the shoulder flexion/extension axis, a case that, along with its variants, achieved the lowest tremor suppression torque/amplitude values.

This control uses state-of-the-art approaches introduced by encoder networks to extract information from changing observations induced by tremors to achieve high-performance tremor suppression. The achieved performances also highlight the need for improved neural network architectures in these algorithms to improve the safety and stability of these control methods, which cannot be bypassed by hybrid traditional learning-based control approaches because the neural network actor’s densely connected architecture can generate values vastly different in concurrent time steps. These generated values cannot be mitigated meaningfully by a traditional proportional-integrative-derivative (PID) controller. This is a future challenge to be addressed due to the limitations of the frequency of control actuation usually present (30–40 Hz) in the actuators.

The results show promise, but the current research is limited to simulation. We mitigate this limitation through domain randomization methods as much as possible. Furthermore, as our training algorithm relies on Markov decision process (MDP) formulation, additional considerations must be made to maintain accurate sensor readings by either state estimation techniques such as Kalman filters (Kalman, 1960) or by incorporating an algorithm that can handle partially observable states. Movements that differ significantly from the reference trajectories used in training may limit the controller’s accuracy. Consequently, future work should include experimental validation on patients and testing with out-of-distribution simulation movements to fully understand how this impacts the controller’s performance. For safety reasons, built-in safety checks, torque limits, and acceleration thresholds should be incorporated into the deployed exoskeleton to further mitigate this problem.

Current applications of the exoskeleton control system extend to real-life rehabilitation exercises, similar to the trajectories present during training.

## 5 Conclusion

This paper proposes a physical simulation-based tremor-suppressing exoskeleton physical simulation framework. The framework is flexible and adaptable to different diseases and characteristics of patients with tremor symptoms. It can also be incorporated with various other dynamic movements. This simulation also proposes an inexpensive and rapid method of validating control algorithm performances. The simulation hyperparameters are included in the supplementary material.

The paper also details the training of a reinforcement learning-based encoder-actor controller. The controller can adapt to personalized interventions in the management of movement disorders. Additionally, the controller can adjust to varying ranges of actuator forces, thereby proposing a viable strategy for tremor suppression.

Experimental results show that the proposed framework can mitigate tremor torques present at the joint axes, and the entire tremor amplitude with tremor propagation is taken into account. The results indicate a substantial decrease in both median and maximum tremor amplitudes.

The control aims to mitigate tremors without interfering with the original movement, not allowing patients to rely too heavily on the exoskeleton during natural motor abilities, thereby not hindering rehabilitation efforts while also minimizing the potential side effects that might arise from prolonged use.

In the future, we intend to deploy the trained exoskeleton control on physical hardware, incorporating sim-to-real techniques into the physical simulation. Furthermore, we aim to validate the performance of this control in a clinical trial setting with patients involved.

## Data Availability

The raw data reference motion recordings in the article will be made available by the authors, without undue reservation. Also the log files of the evaluation process will be made available to ensure the accuracy and transparency of the research. The modified version of the TD7 algorithm is open sourced: (https://github.com/TomasDelaney/A-Deep-Reinforcement-Learning-Enabled-Soft-Exoskeleton-for-Parkinson-s-Patients).
