# Supplementary material for: Learning to suppress tremors: a deep reinforcement learning-enabled soft exoskeleton for Parkinson’s patients
Source: Front Robot AI. 2025 May 21;12:1537470. doi: 10.3389/frobt.2025.1537470 (PMC12133501; doi:10.3389/frobt.2025.1537470)
Supplement: Supplementary file 1 [file DataSheet1.pdf]

Supplementary Material

1 REWARD FUNCTION ABLATION STUDY

Previously we have introduced the reward function, which specifies the agent's behavior as follows:

$$r_t = w_a \cdot r_t^a + w_\mathcal{T} \cdot r_t^\mathcal{T} + w_F \cdot r_t^F + w_{as} \cdot r_t^{as} + w_u \cdot r_t^u \tag{S1}$$

Where r_t^a is the tremor axis reward, $r_t^\mathcal{T}$ is the torque reward, r_t^F is the actuator force reward, r_t^{as} is the action smoothness reward and r_t^u is the unwanted movement reward part. In the following we will investigate how these parts effect the tremor suppression, force use and action smoothness of the exoskeleton. For this we sample 100 episodes of random tremor suppression characteristics for a tremor effecting the shoulder abduction/adduction (SAA) and elbow flexion/extensions (EFE) arm joint axes.

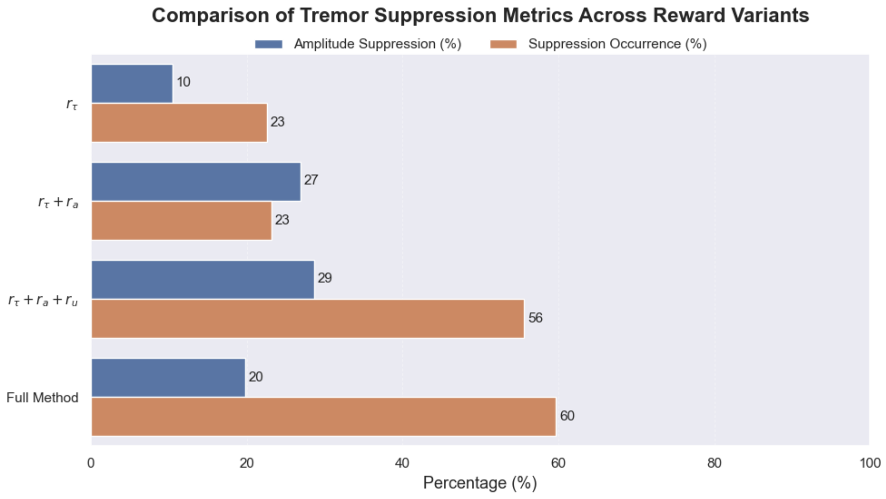

Figure S1: Tremor amplitude suppression percentage and occurrence, with different reward functions.

Figure S1 shows the average tremor suppression percentage and the frequency of suppression time steps across 100 episodes. Relying solely on the tremor torque reward is insufficient for learning effective behavior. Adding the axis reward significantly improves suppression amplitude without affecting its occurrence. Incorporating the unwanted reward component greatly increases the occurrence of suppression. The full method achieves a balance—trading a small reduction in suppression amplitude for higher occurrence and more optimal, safer actuator behavior.

Figure S2 illustrates the average actuator force usage during the same experiments. The reward using only the torque component results in poor learning, with the elbow extender and flexor actuators operating

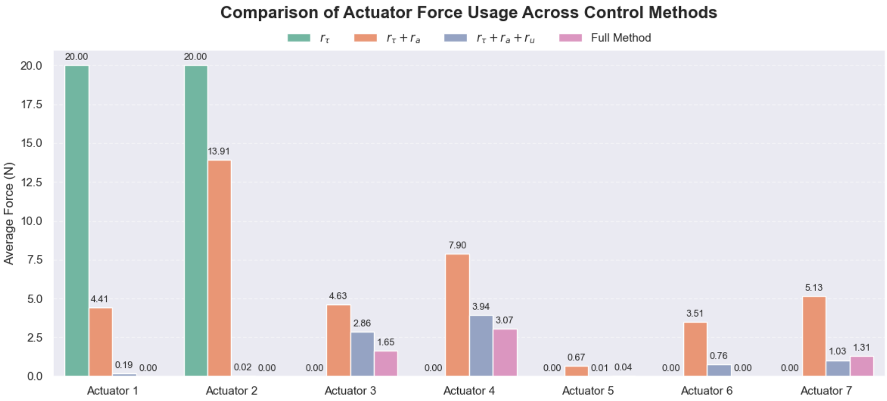

Figure S2: Actuator force usage across different reward functions.

near their maximum limits, while the remaining actuators are largely inactive—indicating inefficient and unsafe control. Incorporating the axis reward leads to more distributed actuator engagement, suggesting improved coordination. The addition of the unwanted reward component substantially reduces overall force output across the exoskeleton, promoting safer actuation. The full method selectively activates only the most effective actuators, suppressing unnecessary force in others to achieve both efficiency and safety.

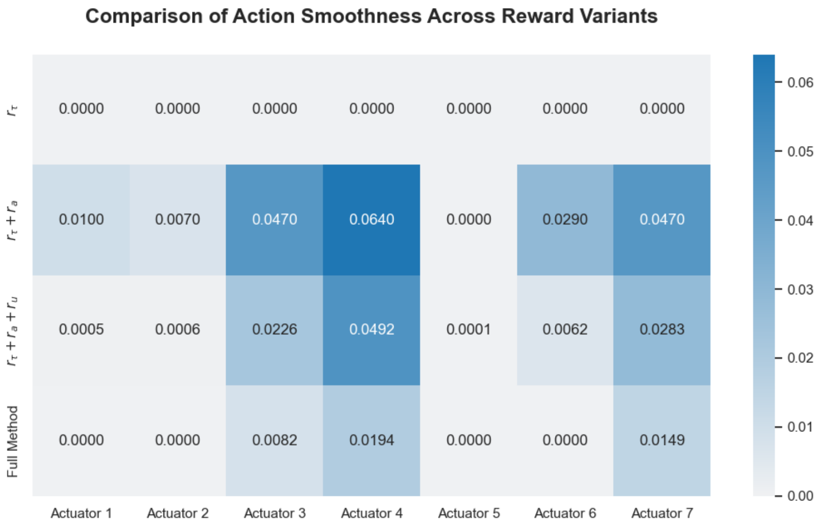

Figure S3: Actuator action smoothness across different reward functions.

Figure S3 displays the action smoothness of each actuator averaged throughout the experiments. The action smoothness is calculated via the following formula:

$$\text{Action Smoothness} = \frac{1}{N}\sum_{t=1}^{N}(a_{t+2} - 2a_{t+1} + a_t)^2 \tag{S2}$$

where a_t denotes the actuator force at time-step t, with lower values indicating smoother transitions in actuator force across consecutive time-steps. From Figure S2, it is evident that relying solely on the tremor torque reward fails to produce meaningful behavior, as actuator forces remain unchanged—specifically, actuators 1 and 2 as observed by Figure S2 and the fact that the action smoothness is 0. Introducing the axis reward enables more dynamic actuation of the motors, evident by the nonzero action smoothness values. The addition of the unwanted reward component improves the overall smoothness of the actions, promoting more efficient control. The complete method achieves the lowest action smoothness values, highlighting its superior safety and energy efficiency.

2 REINFORCEMENT LEARNING HYPERPARAMETERS

Table S1. The neural network structures of the agent and the simulation parameters. In this table the max shoulder actuator force refers to actuators impacting the shoulder joint: actuator 3,4,5,6,7. The max elbow actuation force refers to actuators effecting the elbow joint: actuators 1,2.

	**Hyperparameter**	**Setting**
**Actor network**	Hidden dims	320
	Activation	ReLU
	Learning rate	3e-4
**Critic Network**	Hidden dims	320
	Activation	Elu
	Discount Factor	0.99
**Encoder network**	Hidden dims	300
	Activation	Elu
	Learning rate	3e-4
**Training**	Total training steps	6e6
	Batch size	64 x 8
	Replay buffer size	2.5e5 x 8
	LAP: α	0.4
**Exploration**	Warmup steps	25e3
	Action exploration noise	N(0, 0.1)
	Target policy noise	N(0, 0.2)
**Exoskeleton**	Max actuator force shoulder	40 N
	Max actuator force elbow	20 N

The hyperparameters for the reinforcement learning agent are listed in Table S1. The choices behind the hyperparameter are the following. The actor network usually does not benefit from a deeper or wider architecture, but the amount of hidden dims it has mostly depend on the state observation dimensions of the environment Andrychowicz et al. (2021). Contrary to this we found that since we concatenate the state-embeddings into the network's forward pass it benefits from slightly wider layers. Critic networks have been shown to perform better having wider layers Ota et al. (2020); Bhatt et al. (2024); Nauman et al. (2024). The hidden dimension sizes of the encoder networks have been determined by trial and error.

Regarding batch size, although it has been shown that larger batch sizes can work for reinforcement learning McCandlish et al. (2018), current state-of-the-art algorithms utilize batch sizes of 128 or 256 Fujimoto et al. (2023); Nauman et al. (2024); Bhatt et al. (2024). These parameters are optimal because they are less prone to overfitting and are less computationally straining. For learning rates, we have followed common hyperparameters, which are substantiated by experiments shown in Andrychowicz et al. (2021). For activation functions we used baseline TD7 implementation activation functions: ReLU for the actor network and Elu Clevert et al. (2015) for the critic and encoder networks.

For exploration, we utilize warmup steps to avoid overfitting to early experiences in the replay buffer. We use a slightly larger capacity replay buffer which can be beneficial according to Fedus et al. (2020). We also divide the buffer into sub-buffers and uniformly sample 64 transition tuples to avoid biased training. For the exoskeleton actuator forces, we selected a generic range that ensures sufficient capability without requiring overly precise actuators.

3 SIMULATION HYPERPARAMETERS

Table S2. The human-exoskeleton physical simulation joint angle properties Zwerus et al. (2019), Gill et al. (2020).

Joint name	Maximum position (deg)	Minimum Position (deg)
EFE	150	-2
FPS	80	-87.7
SAA	0	-151.5
SFE	160.5	-40
SEIR	20	-58.8

Table S3. The characteristics of the used sine waves approximating Parkinson's tremors.

Name	Frequency (Hz)	Amplitude (dB)
First harmonic	4 - 6	-5 - 0
Second harmonic	8 - 10	-10 - -5

Table S4. The maximum joint torques for the dynamic movements for each joint axis Günzkofer et al. (2012), Otis et al. (1990).

Joint name	Maximum torque values (Nm)
EFE	25
FPS	50
SAA	50
SFE	80
SEIR	40

Table S5. Human anatomical parameters used in the simulation. The upper and lower arm weights are calculated using the ratios defined in Plagenhoef et al. (1983).

Parameter	Value
Weight	81.5 kg
Humerus Length	0.4 m
Forearm Length	0.4 m
Humerus Radius	0.05 m
Forearm Radius	0.05 m
Hand Length	0.05 m

Table S6. The Denavit and Hartenberg parameters used for the end effector point calculations. Where l_u is the length of the humerus and l_f is the length of the forearm Bertomeu-Motos et al. (2018).

**Joint name**	**theta**	**d**	**a**	**alpha**
$q1$	q_1	0	0	$\pi/2$
$q2$	q_2	0	0	$\pi/2$
$q3$	q_3	l_u	0	$-\pi/2$
$q4$	q_4	0	0	$\pi/2$
$q5$	q_5	l_f	0	$\pi/2$
$q6$	q_6	0	0	$\pi/2$
$q7$	q_7	0	0	$\pi/2$

Table S7. Tremor propagation parameters used in the joint axes angle calculations Davidson and Charles (2017).

Inertia (kg m^2)							
	SFE	SAA	SIER	EFE	FPS	WFE	WRUD
SFE	0.269	0	0	0.076	0	0	-0.014
SAA	0	0.196	0.083	0	-0.002	0.009	0
SIER	0	0.083	0.079	0	0	0.011	0
EFE	0.076	0	0	0.076	0	0	-0.012
FPS	0	-0.002	0	0	0.002	0	0
WFE	0	0.009	0.011	0	0	0.003	0
WRUD	-0.014	0	0	-0.012	0	0	0.003
Damping (Nms/rad)							
	SFE	SAA	SIER	EFE	FPS	WFE	WRUD
SFE	0.756	0.184	0.020	0.187	0	0	0
SAA	0.184	0.383	0.267	0	0	0	0
SIER	0.020	0.267	0.524	0	0	0	0
EFE	0.187	0	0	0.607	0	0	0
FPS	0	0	0	0	0.021	0.001	0.008
WFE	0	0	0	0	0.001	0.028	-0.003
WRUD	0	0	0	0	0.008	-0.003	0.082
Stiffness (Nm/rad)							
	SFE	SAA	SIER	EFE	FPS	WFE	WRUD
SFE	10.80	2.626	0.279	2.670	0	0	0
SAA	2.626	5.468	3.821	0	0	0	0
SIER	0.279	3.821	7.486	0	0	0	0
EFE	2.670	0	0	8.670	0	0	0
FPS	0	0	0	0	0.756	0.018	0.291
WFE	0	0	0	0	0.018	0.992	-0.099
WRUD	0	0	0	0	0.291	-0.099	2.920

4 ADDITIONAL TREMOR SUPPRESSION METRICS

Tables providing additional more in depth tremor suppression information for each arm joint axes and dynamic movements are provided as additional supplementary material tables.

REFERENCES

Andrychowicz, M., Raichuk, A., Stańczyk, P., Orsini, M., Girgin, S., Marinier, R., et al. (2021). What matters in on-policy reinforcement learning? a large-scale empirical study. In *ICLR 2021-Ninth International Conference on Learning Representations*

Bertomeu-Motos, A., Blanco, A., Badesa, F. J., Barios, J. A., Zollo, L., and Garcia-Aracil, N. (2018). Human arm joints reconstruction algorithm in rehabilitation therapies assisted by end-effector robotic devices. *Journal of neuroengineering and rehabilitation* 15, 1–11

Bhatt, A., Palenicek, D., Belousov, B., Argus, M., Amiranashvili, A., Brox, T., et al. (2024). Crossq: Batch normalization in deep reinforcement learning for greater sample efficiency and simplicity. In *International Conference on Learning Representations (ICLR)*

Clevert, D.-A., Unterthiner, T., and Hochreiter, S. (2015). Fast and accurate deep network learning by exponential linear units (elus). *arXiv preprint arXiv:1511.07289*

Davidson, A. D. and Charles, S. K. (2017). Fundamental principles of tremor propagation in the upper limb. *Annals of biomedical engineering* 45, 1133–1147

[Dataset] Fedus, W., Ramachandran, P., Agarwal, R., Bengio, Y., Larochelle, H., Rowland, M., et al. (2020). Revisiting fundamentals of experience replay

Fujimoto, S., Chang, W.-D., Smith, E. J., Gu, S. S., Precup, D., and Meger, D. (2023). For sale: State-action representation learning for deep reinforcement learning. *arXiv preprint arXiv:2306.02451*

Gill, T. K., Shanahan, E. M., Tucker, G. R., Buchbinder, R., and Hill, C. L. (2020). Shoulder range of movement in the general population: age and gender stratified normative data using a community-based cohort. *BMC musculoskeletal disorders* 21, 1–9

Günzkofer, F., Bubb, H., and Bengler, K. (2012). Maximum elbow joint torques for digital human models. *International Journal of Human Factors Modelling and Simulation* 3, 109–132

McCandlish, S., Kaplan, J., Amodei, D., and Team, O. D. (2018). An empirical model of large-batch training. *arXiv preprint arXiv:1812.06162*

Nauman, M., Ostaszewski, M., Jankowski, K., Miłoś, P., and Cygan, M. (2024). Bigger, regularized, optimistic: scaling for compute and sample-efficient continuous control. In *Advances in Neural Information Processing Systems*

Ota, K., Oiki, T., Jha, D., Mariyama, T., and Nikovski, D. (2020). Can increasing input dimensionality improve deep reinforcement learning? In *International conference on machine learning* (PMLR), 7424–7433

Otis, J. C., Warren, R. F., Backus, S. I., Santner, T. J., and Mabrey, J. D. (1990). Torque production in the shoulder of the normal young adult male: the interaction of function, dominance, joint angle, and angular velocity. *The American journal of sports medicine* 18, 119–123

Plagenhoef, S., Evans, F. G., and Abdelnour, T. (1983). Anatomical data for analyzing human motion. *Research quarterly for exercise and sport* 54, 169–178

Zwerus, E. L., Willigenburg, N. W., Scholtes, V. A., Somford, M. P., Eygendaal, D., and van den Bekerom, M. P. (2019). Normative values and affecting factors for the elbow range of motion. *Shoulder & elbow* 11, 215–224
